# 

**Published:** 1902-01-25

**Authors:** 


### NOTICE TO CORRESPONDENTS.

All MSS., Letters, Books for Review, and other matters intended for the Editor should be addressed The Editor, "The Hospital," The Hospital Building, 28 & 29 Southampton Street, Strand, W.C.

The Editor cannot undertake to return rejected MS., even when accompanied by stamped directed envelope.

**Notice to Advertisers and Subscribers.**

All Advertisements, Orders for Copies of The Hospital, and Business Communications should be addressed to **THE MANAGER (Not to the Editor),** The Hospital, 28 & 29 Southampton Street, Strand, London, W.C.

The Cost of Subscription, post free, is as follows:—Per week, 2½d.; per quarter, 2s. 8d.; per half-year, 5s. 3d.; per annum, 10s. 6d. Foreign:—Per annum, 15s. 2d., payable, in all cases, in advance.

**NOTICE.—Vol. XXX. of "The Hospital" (April 1901, to September 1901), in handsome cloth binding can now be obtained at the office of this Journal, price, 7s. 6d. Cases for binding the Numbers contained in this Volume 1s. 6d. Index to Vol. XXX., 2½d., post free.**

**The Monthly Part for January is now ready. Price 6d., by post 8d.**

### BOOKS RECEIVED.

J. B. LIPPINCOTT CO.

"An Experimental and Clinical Research into Certain Problems relating to Surgical Operations." (Alvarenga Prize Essay.) By George W. Crile, A.M., M.D., Ph.D.

"Pediatrics; the Hygienic and Medical Treatment of Children." By Thomas Morgan Rotch, M.D. Third Edition.

LONGMANS, GREEN, AND CO.

"Quain's Dictionary of Medicine." By various writers. Third Edition. Edited by H. Montague Murray, M.D., F.R.C.S., assisted by John Harold, M.B., B.Ch., B.A.O., and W. Cecil Bosanquet, M.A., M.D., M.R.C.P.

### SCRAPS AND GLEANINGS.

THE King has sent a gift of 20 pheasants to Queen Charlotte's Hospital, Marylebone Road.

THE Queen has sent her annual subscription of £5 to Queen Charlotte's Hospital, of which she is patron, and the Princess of Wales (vice-president) has sent a donation of £5.

THE Queen has sent her annual subscription of five guineas to the funds of the British Home and Hospital for Incurables, at Streatham, which was the first institution in England to receive the patronage of Her Majesty.

THE Princess of Wales has also sent a donation of £5 to the same charity.

AN interesting article on the Open-air Treatment of Consumption, with illustrations of the New Liverpool Sanatorium and Mount Vernon Hospital at Hampstead, appears in the *Graphic* for January 11th.

BY direction of Her Majesty Queen Alexandra, a cheque for £9 1s. 1d. has been forwarded to the Secretary of the British Home and Hospital for Incurables, Streatham, as the result of a further sale of Canon Fleming's Sermon "Recognition in Eternity."

THE sixty-third annual general meeting of the Newsvendors Benevolent and Provident Institution will be held at the Memorial Hall, Farringdon Street, London, E.C., on Tuesday evening, February 11th, 1902, the Right Hon. Lord Glenesk in the chair.

As a result of the dramatic performance arranged by the Misses Bendon in aid of the Jews' Deaf and Dumb Home at Wandsworth, the handsome sum of £520 will be handed over to this institution. The performance, which consisted of a representation of H. V. Esmond's play, "One Summer's Day," was held at St. George's Hall.

### The Student of Medicine.

### APPOINTMENTS.

**A. Barber,** M.D.Brux., L.R.C.S.I., appointed Government Medical Officer and Vaccinator at Penrith, New South Wales. **H. C. Beasley,** L.R.C.P., L.R.C.S.I., appointed District Medical Officer of the Staines Union. **G. H. Bedford,** L.S.A., appointed District Medical Officer of the Rothbury Union. **E. E. Bowden,** M.R.C.S.Eng., L.S.A., appointed Certifying Factory Surgeon for the Warrington District. **Alfred Tennyson Brown,** M.B., C.M., appointed Medical Officer of Health for the Horbury Urban District Council. **Herbert Carre-Smith,** L.R.C.P.Lond., M.R.C.S.Eng., appointed Examining Medical Officer for the Kensington District to the Morley Convalescent Home, St. Margaret's Bay, Dover; and to the Alfred Bevan Memorial Home for Men, Women, and Children, Sandgate. **R. F. Castle,** M.B., B.C.Camb., appointed District Medical Officer of the Barnsley Union. **William H. Cooke,** F.R.C.S.E., M.R.C.S., L.R.C.P., appointed Assistant Surgeon to the Royal United Hospital, Bath. **H. S. Cooper,** M.R.C.S., L.R.C.P.Lond., appointed District Medical Officer of the Peterborough Union. **A. Cutfield,** B.A.Cantab., M.R.C.S.Eng., appointed Certifying Factory Surgeon for the Urban District of Ross and the Rural Districts of Ross and Whitchurch, county Hereford. **C. de P. D'Amico,** M.D.Malta, M.R.C.S.Eng., L.R.C.P.Lond., appointed an Assistant Medical Officer in the Hospital Service of the Metropolitan Asylums Board. **Henry George Dickman,** M.B., C.M.Edin., appointed Medical Officer of Health for the Diss Urban District Council, Norfolk, and Medical Officer and Public Vaccinator to the No. 5 District (Diss) of the Depwade Union, Norfolk. **W. B. Dove,** M.B., B.C.Camb., appointed District Medical Officer of the Hendon Union. **G. Fleming,** M.B., C.M.Edin., appointed District Medical Officer of the Martley Union. **A. W. Graham,** M.B.Lond., M.R.C.S.Eng., appointed Medical Officer of Health for the District of Beaconsfield and Dalrymple, Tasmania. **T. P. Greenwood,** L.R.C.P.Edin., M.R.C.S.Eng., appointed Certifying Factory Surgeon for the Stamford District of Lincolnshire and the Easton-on-the-Hill District of Northampton. **Lauder Hamilton,** M.B., Ch.B.Aberd., appointed Resident House Physician at Chester General Infirmary. **R. H. Hemsted,** M.R.C.S., L.R.C.P.Lond., appointed Certifying Factory Surgeon for the Rural Districts of Whitchurch and Kingsclere, county Hants. **C. M. Hewer,** F.R.C.S.Eng., appointed Certifying Factory Surgeon for the Tarporley District of Cheshire. **B. B. Hosford,** M.B., B.S.R.U.I., appointed District Medical Officer of the Bradfield Union, *vice* R. Cox, M.D.St And. **C. C. Lall,** M.B., B.Ch.Edin., appointed an Assistant Medical Officer in the Hospital service of the Metropolitan Asylums Board. **Frederick J. McCann,** M.D.Edin., F.R.C.S.Eng., M.R.C.P. Lond., appointed Physician to In-patients at the Samaritan Free Hospital for Women, London. **J. G. Macindoe,** M.B., C.M.Edin., appointed Medical Officer of the Workhouse of the Torrington Union. **W. Mackirdy,** L.R.C.P., L.R.C.S.Edin., L.F.P.S.Glasg., appointed Medical Officer for the Stilton District of the Peterborough Union. **J. S. Mellish,** M.R.C.S. L.R.C.P.Lond., appointed District Medical Officer of the Tetbury Union. **W. C. C. Muir,** M.B., Ch.M.Glasg., appointed Officer of Health for the shire of Alberton, Victoria. **Thos. C. Penfold,** M.B., C.M.Edin., appointed Medical Officer of Health for the Hexham Rural District. **T. A. S. Plowman,** M.R.C.S., L.R.C.P.Lond., appointed District Medical Officer of the Taunton Union. **G. I. T. Stewart,** M.B., M.S.Aberd., appointed Assistant Medical Officer to the Brentford Union Infirmary. **J. E. F. Stewart,** M.B., Ch.M.Glasg., appointed Officer of Health to the Bayswater Local Board of Health, West Australia. **Henry G. Terry,** M.R.C.S., F.R.C.S.E., appointed Surgeon to the Royal United Hospital, Bath. **C. Thackray,** M.R.C.S., L.R.C.P.Lond., appointed Third Assistant Medical Officer of the Bethnal Green Infirmary. **Robert F. S. Wallace,** M.R.C.S., L.R.C.P.Lond., appointed Medical Officer of Health for the Skegness Urban District.

